# Surface Electromyographic Responses During Rest on Mattresses with Different Firmness Levels in Adults with Normal BMI

**DOI:** 10.3390/s25010014

**Published:** 2024-12-25

**Authors:** Xiaohong Hu, Yuhong Gao, Keyang Liu, Luyao Xiang, Bin Luo, Li Li

**Affiliations:** 1 College of Material Science and Technology, Beijing Forestry University, Beijing 100083, China; 2 School of Management, Yulin University, Yulin 719000, China; 3 College of Fashion and Design Art, Sichuan Normal University, Chengdu 610101, China

**Keywords:** mattress firmness, sEMG, BMI, anthropometric measurements

## Abstract

Mattress firmness is a critical factor influencing sleep quality. This study investigates the effects of time, mattress firmness, and anthropometric parameters on surface electromyographic (sEMG) activity during supine rest. Eleven participants were analyzed, and the *RMS* values for lower back sEMG on three mattresses were measured as 8.37 ± 0.85 μV, 8.43 ± 1.06 μV, and 7.77 ± 1.15 μV during the final five minutes of testing, showing no statistical significance. Similarly, no significant differences in electromyographic activity were observed across different time periods. Anthropometric parameters such as BMI, waist circumference (WC), and waist-to-hip ratio (WHR) significantly influenced sEMG activity on firm mattresses. Higher BMI, waist circumference, and waist-to-hip ratio were negatively correlated with *RMS* and *iEMG* values on firm mattresses, suggesting that firmer mattresses are better suited for individuals with higher BMI, larger waist circumference, and a more balanced distribution of body weight between the upper and lower body. The medium-firm mattress had the lowest discomfort scores, indicating its broader adaptability. These findings provide a foundation for designing ergonomically optimized, personalized mattresses to improve sleep quality across diverse populations.

## 1. Introduction

Sleep is a critical factor in maintaining both physical and mental health [1,2]. High-quality sleep significantly enhances learning and memory capabilities and optimizes logical decision-making and problem-solving abilities through prefrontal cortex regulation [3,4,5]. Furthermore, during sleep, emotional circuits in the brain undergo recalibration, which contributes positively to mental health [6,7].

As a primary determinant of sleep quality, bedding—particularly mattress design—has gained increasing attention in recent years. An ideal mattress must provide both comfort and adequate support to accommodate the physiological characteristics and sleeping habits of individuals with diverse body types. An inappropriate sleeping surface can lead to musculoskeletal issues, such as chronic pain and lower back pain, which severely compromise sleep quality [8,9]. However, current research on mattress evaluation methodologies remains relatively limited, highlighting the need to develop multidimensional assessment systems to guide mattress design in a more systematic and scientific direction.

The assessment of mattresses typically focuses on three key aspects: subjective comfort [10,11], pressure distribution at the body–mattress interface [12,13,14], and spinal support [15,16,17]. Comfort is defined as a pleasant state of relaxation or ease, reflecting a combination of physiological and psychological satisfaction. In contrast, discomfort is often associated with biomechanical factors and fatigue, representing negative perceptions of the physical environment [18,19,20].

Research often approaches comfort analysis by focusing on discomfort or pain. For example, tools such as the Visual Analog Scale (VAS) [11] are used to evaluate the effects of mattress firmness, material, and structure on outcomes like lower back pain, shoulder pain, and lumbar stiffness [21,22]. Additionally, subjective evaluations that consider self-reported sleep quality were also used [23,24]. However, subjective methods face notable limitations, including variability in individual perceptions, stringent experimental requirements, and susceptibility to external influences, which challenge their reliability and reproducibility.

Pressure distribution at the body–mattress interface is another widely used mattress evaluation method [13,14,25,26]. Key metrics include average pressure, peak pressure, and contact area. Lower interface pressures and larger contact areas are generally considered to be associated with improved comfort, a hypothesis based on biomechanical principles of skin tissue comfort. Studies have shown that interface pressures above 30 mmHg can restrict blood flow and cause tissue ischemia [27], especially in long-term bedridden patients, which increases the risk of pressure ulcers. Therefore, reducing interface pressure is a key focus in pressure ulcer prevention [28,29]. However, in healthy individuals, the interaction between the body and the mattress is dynamic, raising concerns about whether methods designed to prevent pressure ulcers are fully applicable to studying mattress comfort.

Spinal alignment assessment is another critical method in mattress research. This approach typically uses the natural spinal alignment observed in a standing position as an ideal reference, evaluating how well different mattresses maintain this alignment during sleep [17]. Specific techniques include marking points along the spine to assess alignment in a lateral sleeping position [26], placing movable rulers beneath the mattress to measure regional deflection [30], and using 3D scanning technology for comprehensive spinal shape analysis [31]. These methods aim to determine the mattress’s effectiveness in supporting proper spinal alignment.

Additionally, some studies have used polysomnography (PSG) and other physiological recording techniques to investigate the effects of mattress firmness and comfort on sleep quality. However, due to limitations in experimental design and subject selection, these studies have not yet produced consistent or significant findings [32,33]. The impact of mattress characteristics on sleep remains unclear.

Research on the relationship between mattresses and electromyographic (sEMG) signals is still limited, with inconsistent results. For example, Lee et al. identified the optimal combination of mattress topper firmness and thickness through a combined analysis of sEMG and pressure distribution, revealing significant effects of firm mattress toppers on trapezius and biceps femoris sEMG activity [26]. In contrast, Lahm and Iaizzo found no significant differences in sEMG activity for the trapezius and lumbar paraspinal muscles across different mattress firmness levels [34]. These discrepancies highlight variations in experimental designs and evaluation methodologies, underscoring the need for further research to develop more reliable systems for assessing mattress comfort.

Reducing interface pressure effectively alleviates compromised blood circulation caused by excessive pressure, thereby enhancing the comfort perception in skin tissues [19]. However, excessively low interface pressure may indicate insufficient mattress support, which can compromise spinal alignment stability and overall body support [30]. Currently, the scientific foundation for using pressure as a key metric for mattress comfort evaluation requires further investigation.

Regarding spinal alignment, most existing evaluation methods use the natural spinal posture observed during standing as the ideal reference. However, spinal alignment in a lying position differs significantly from that in a standing position. Moreover, traditional spinal assessment techniques often rely on photographic comparison, limiting analysis primarily to lateral sleeping postures [34] while leaving spinal alignment in the supine position largely unstudied.

In light of these limitations, this study uses surface electromyography (sEMG) as a core metric for evaluating mattress comfort. Exploring the effects of mattress firmness on sEMG signals can reveal the biomechanical interactions between the human body and the mattress, broadening the scope of mattress design and evaluation and providing a scientific basis for personalized mattress development. Additionally, although modern smart mattresses often feature adjustable firmness, such adjustments are primarily based on short-term subjective feedback or simplistic algorithms, lacking dynamic monitoring and feedback on the body’s long-term physiological state. With the rapid advancement of wearable technology and artificial intelligence, future smart mattresses could leverage sEMG monitoring to enable automated adjustments. However, current research on sEMG responses under varying mattress conditions remains limited, and a comprehensive understanding of these interactions is necessary to advance the field.

This study focuses on three primary research areas. First, it examines the temporal patterns of sEMG signals from the shoulder, upper back, lumbar, and gluteal muscles while resting on soft, medium, and hard mattresses. Theoretically, insufficient or excessive support from overly soft or overly firm mattresses may cause localized muscle fatigue, particularly in the lumbar region. This research aims to identify these temporal patterns and underlying mechanisms. Second, it conducts a comparative analysis of sEMG activity across mattress firmness levels, comparing the effects of soft, medium, and firm mattresses on sEMG activity during the same resting period. This analysis seeks to identify significant differences in sEMG responses associated with changes in mattress firmness. Lastly, this study explores the relationship between sEMG signals and anthropometric parameters, such as body weight, height, and body shape. It aims to understand the interplay between individual body characteristics and mattress firmness, establishing a theoretical basis for personalized mattress design.

This study comprehensively evaluates mattress adaptability through a multidimensional analysis of sEMG signals in a resting state. Unlike previous research that focused solely on the direct impact of mattress firmness on sEMG activity, this study also explores the relationship between individual anthropometric parameters and sEMG changes, providing a theoretical foundation for the future development of more personalized bedding designs and smart mattresses.

## 2. Materials and Methods

### 2.1. Subjects

This study recruited healthy right-handed participants from Beijing Forestry University, aged 18–30, with no history of musculoskeletal disorders or spinal surgery. All participants were undergraduate or graduate students with a BMI between 18.5 and 24.9. To prevent muscle fatigue, participants were instructed to avoid intense exercise for 24 h before the experiment and ensure they obtained at least 7 h of sleep. Additionally, sleep diaries were used to assess their sleep status. To minimize variability in fatigue levels, participants were required to arrive at the laboratory before 8:30 AM, and the experiment officially began at 9:00 AM. This standardized start time helped reduce muscle fatigue errors that could arise from individual differences [35]. The protocol was approved by the Human Study Ethics Committee of Beijing Forestry University (Approval No: BJFUPSY-2024-032), and all participants provided written informed consent.

As seen in Table 1, a total 11 participants (4 males and 7 females) were included in the study. The participants had an average age of 23.09 ± 1.86 years, an average height of 1.72 ± 0.81 m, an average weight of 63.64 ± 7.15 kg, and an average BMI of 21.41 ± 1.42.

### 2.2. Instrument and Experiment Environment

The experiment utilized three mattresses with varying firmness levels (soft, medium, and firm), all made of polyurethane foam, each measuring 1200 mm × 1950 mm × 100 mm. Firmness was assessed using the HANDPI LX-F sponge hardness tester (HANDPI LX-F, Shenzhen, China); the hardness meter was gently placed on the specimen at the center. The reading is taken within one second after the probe of the hardness meter makes stable contact with the sample. To improve measurement accuracy, five measurements are taken at least 25 mm apart on different areas of each specimen. The average of these readings is taken as the hardness value of the material. The hardness values of the four mattresses are 32.6 HA, 64.6 HA, and 92 HA, respectively. The laboratory environment was controlled to maintain an indoor temperature of 21 ± 2 °C, relative humidity of 45 ± 5%, and a noise level of ≤50 db.

Surface electromyography (sEMG) measured muscle activity across mattress types. An 8-channel sEMG system (Kingfar Technology Inc., Beijing, China) recorded signals at 1000 Hz with a band-pass filter of 10–500 Hz. Sensors, each comprising two measurement electrodes (Ag/AgCI) and a reference electrode, transmitted signals to the ErgoLAB (Kingfar Technology Inc., Beijing, China) platform for preprocessing and feature extraction.

### 2.3. Procedure

**Figure 1:**
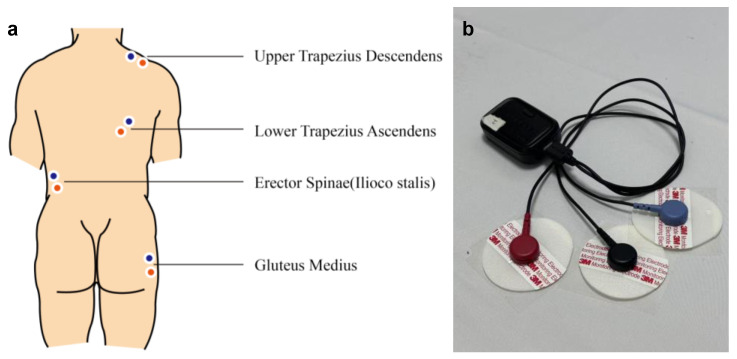
(**a**): Diagram of EEG electrode placement; (**b**): Picture of the sEMG recording equipment.

All participants arrived at the lab at 8:30 AM. After providing informed consent and completing anthropometric measurements, sEMG devices were applied. Electrode placement followed The SENIAM Project guidelines [36] and targeted four muscles: the upper trapezius, lower trapezius, erector spinae–iliocostalis, and gluteus medius, representing the shoulder, upper back, lower back, and hip, respectively (see Figure 1a). Disposable bipolar Ag/AgCl sEMG electrodes, each with a diameter of 50 mm, were placed on the prominent areas of the four tested muscles, with the centers of the two electrodes positioned 25 mm apart (see Figure 1b). For the upper trapezius, the electrodes were positioned at the midpoint of the line between the acromion and the C7 spinous process, oriented along this line. For the lower trapezius, the electrodes were positioned at two-thirds of the distance along the line from the trigonum spinae to the T8 spinous process, oriented in the direction of the line connecting T8 and the acromion. For the erector spinae–iliocostalis, the electrodes were placed one finger width medial from the line from the posterior spina iliaca superior to the lowest point of the lower rib, at the level of L2, oriented along the line connecting the posterior spina iliaca superior and the lowest point of the lower rib. For the gluteus medius, the electrodes were positioned at the midpoint (50%) of the line connecting the crista iliaca to the trochanter, oriented along the same line. The skin was carefully cleansed with alcohol to minimize signal interference. Reference electrodes were positioned on non-muscular areas to minimize signal interference.

This study aimed to evaluate the muscle activity levels during the resting state; therefore, maximum voluntary contraction (MVC) measurements were not conducted. MVC tests are primarily used to quantify the maximum activity of muscles during active contraction. However, this study focused on baseline muscle activity in the resting state, avoiding potential interference with the muscle condition caused by MVC operations to ensure the accuracy of resting-state electromyography (sEMG) data [37,38]. Participants lay supine on the test mattress for 27 min, with data recorded during the central 25 min. A Tekscan pressure mapping system (CONFORMat, Tekscan, Inc., Norwood, MA, USA) was placed under subjects to record real-time pressure distributions.

The mattress order was randomized to prevent sequence bias, with a 15 min break between trials. During breaks, participants were required to engage in low-intensity activities, such as walking, to reduce fatigue. Mattress discomfort was rated using the Borg CR10 scale [39], ranging from 0 (no discomfort) to 10 (extreme discomfort), with ratings provided for four body regions: shoulder, upper back, lower back, and hip. Participants were briefed on the scale before the experiment began.

### 2.4. Measurements

In this study, we used an integrated device and signal processing platform (Kingfar Technology Inc., Beijing, China) that performs filtering and data normalization to ensure the quality of the EMG signals. The signals were sampled at 1000 Hz, and a series of filters were applied to remove unwanted noise and interference. Wavelet denoising was used to eliminate white noise, while band-pass filtering was applied with a frequency range of 10 to 500 Hz to capture the relevant signal components. A band-stop filter set at 50 Hz was used to remove power line interference. Following the filtering process, the signal amplitude was smoothed using a moving root mean square (*RMS*) filter with a window length of 100 ms.

In the time domain, *RMS* and integrated EMG (*iEMG*) were calculated. *RMS* values reflected average muscle activity: increasing trends indicated higher activation while decreasing trends suggested relaxation. *iEMG* quantified cumulative muscle activity: higher values indicated sustained low-level activation or incomplete relaxation, while lower values suggested reduced activity [40].

For frequency-domain analysis, median frequency (*MF*) represented the central frequency of muscle discharges. An increase in *MF* suggested more uniform muscle activity and less fatigue, while a decrease indicated worsening fatigue. Mean power frequency (*MPF*) describes the average frequency content. Higher *MPF* values indicated an increase in high-frequency components, often linked to less fatigue, while lower values suggested greater fatigue.

The formulas for *RMS* (1), *iEMG* (2), *MF* (3), and *MPF* (4) are provided below:

(1) $$RMS=\sqrt{\frac{1}{N}\sum_{i=1}^{N}{EMG\left(i\right)}^{2}}$$

(2) $$iEMG=\sum_{i=N_{2}}^{N_{1}}|EMG(i)|$$

(3) $$\sum_{i=1}^{MDF}P\left(f_{i}\right)=\sum_{i=MDF}^{N}P(f_{i})$$

(4) $$MPF=\frac{\sum_{n=1}^{N}f_{n}\cdot PSD\left(f_{n}\right)}{\sum_{N=1}^{N}PSD\left(f_{n}\right)}$$

*i*: The sample index, where *i* = 1, 2, …, *N*.

*N*: The number of samples in the moving average window.

*EMG*(*i*): The *i*-th sample of the *EMG* signal.

*f*: Frequency, used in power spectral density (*PSD*) calculations.

*PSD*(*f*): Power spectral density of the *EMG* signal at frequency *f*.

### 2.5. Statistical Analysis

To ensure sufficient statistical power for this study, the minimum sample size was calculated using GPower software 3.1.9.7 (Franz Faul, Universitat Kiel, Germany). The calculation was based on the following parameters: an effect size of medium magnitude (Cohen’s *f* = 0.25), three measurement conditions corresponding to the three different mattress types, a correlation of 0.5 between repeated measures (assumed to represent a moderate correlation between conditions), a statistical power of 0.80, and a significance level set at 0.05. Based on these parameters, GPower determined that the required minimum sample size is 8 participants. Given that 11 participants were included in this study, the sample size is sufficient to conduct the analysis and achieve the desired statistical power to detect a medium effect size.

We conducted repeated measures ANOVA to analyze pressure data (peak pressure, average pressure, and contact area) at the human–mattress interface across three mattress types. Muscle activation levels over five time intervals (0–5, 5–10, 10–15, 15–20, and 20–25 min) were also evaluated using repeated measures ANOVA for soft, medium, and firm mattresses. Additionally, sEMG values (*RMS* and *MF*) during the initial (0–5 min) and final (20–25 min) periods were compared to examine changes in muscle activation across mattress firmness conditions. Finally, discomfort ratings were analyzed using repeated measures ANOVA to assess subjective discomfort across the three mattress types.

The Shapiro–Wilk test was used to assess normality, and Mauchly’s test was applied to evaluate the assumption of sphericity. When Mauchly’s test indicated a violation of sphericity (*p* < 0.05), multivariate analysis, specifically Pillai’s Trace, was employed to evaluate the effects. Significant main effects were further analyzed using Newman–Keuls post hoc tests, with the significance level set at *p* < 0.05.

Additionally, Pearson correlation analysis was conducted to explore relationships between anthropometric parameters and sEMG indices. The normality of the data was confirmed prior to analysis to satisfy the requirements for Pearson correlation. The correlation coefficient ranged from −1 to 1, with values closer to 1 indicating stronger relationships between variables. A significance level of *p* < 0.05 was applied to all correlation analyses.

## 3. Results

### 3.1. Effect of Mattress Firmness on Pressure Distribution

A repeated measures analysis of variance (ANOVA) was conducted on the pressure distribution parameters—peak pressure, average pressure, and contact area—under the three mattress conditions.

**Figure 2:**
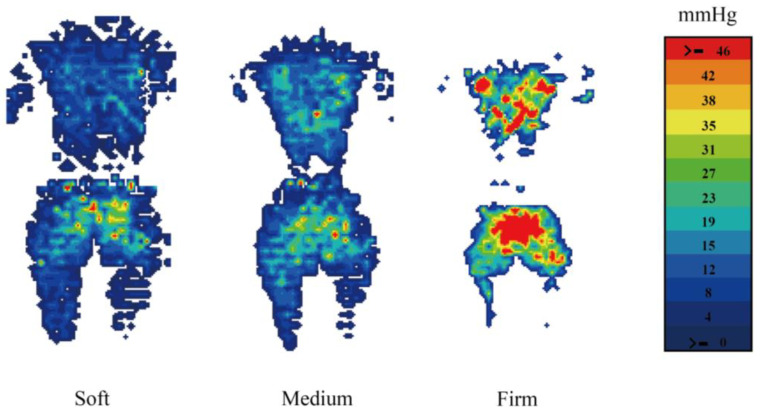
Pressure map on mattresses with different firmness levels of one subject.

As shown in Table 2, as mattress firmness increased, the average pressure significantly increased while the contact area significantly decreased. There was no significant difference in peak pressure between the soft and medium-firm mattresses, but both were significantly lower than the firm mattress. Figure 2 presents the pressure distribution map of one participant across the three mattress types, which corresponds to the data in Table 2. With increasing mattress firmness, the contact area progressively decreased while the red pressure zones (i.e., high-pressure areas) expanded.

### 3.2. Effect of Mattress Firmness on Surface Electromyographic Changes over Time

To investigate temporal patterns of muscle activation across different mattress firmness levels, the root mean square (*RMS*) and mean frequency (*MF*) values of surface electromyographic (sEMG) data were analyzed at 5 min intervals: 0–5, 5–10, 10–15, 15–20, and 20–25 min.

**Figure 3:**
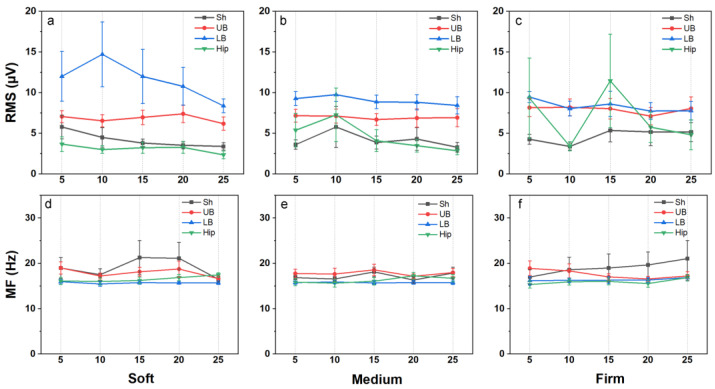
Electromyographic changes over time under different mattress firmness levels. (**a**–**c**) root mean square (*RMS*) changes over time under soft, medium, and firm mattresses. (**d**–**f**) Mean frequency (*MF*) changes over time under soft, medium, and firm mattresses. Note: Sh: Shoulder (Upper Trapezius Descendens); UB: Upper Back (Lower Trapezius Descendens); LB: Lower Back (Erector Spinae, Iliocostalis); Hip: Hip (Gluteus Medius). The x-axis represents successive 5–minute intervals: 0–5 min, 5–10 min, 10–15 min, 15–20 min, and 20–25 min. All data are presented as mean ± SEM, *n* = 11.

As shown in Figure 3, average *RMS* values of lumbar muscles (LB) gradually decreased on the soft mattress, while the other muscle groups showed minimal changes. On the medium-firm mattress, *RMS* values remained stable across all body regions. However, on the firm mattress, average *RMS* values for gluteal muscles exhibited greater fluctuations over time. Despite these variations, none of the changes in *RMS* values across the three mattress conditions were statistically significant.

Similarly, *MF* values showed no significant differences at any time point under soft, medium-firm, or firm mattress conditions, indicating the minimal impact of mattress firmness on muscle frequency characteristics over time. Overall, these findings suggest that the duration spent in the supine position has a limited influence on muscle activation patterns.

### 3.3. Correlation Between Participant Body Parameters and sEMG Data

We calculated the Pearson correlations between anthropometric parameters (height, weight, BMI, waist circumference, hip circumference, and waist-to-hip ratio) and muscle activation indicators of the upper and lower back, including *RMS*, *iEMG*, *MF*, and *MPF*. The results were adjusted for multiple comparisons using the False Discovery Rate (FDR) correction, specifically employing the Benjamini-Hochberg (BH) method. The findings indicated no significant correlations between anthropometric parameters and muscle activation indicators in the upper back.

**Figure 4:**
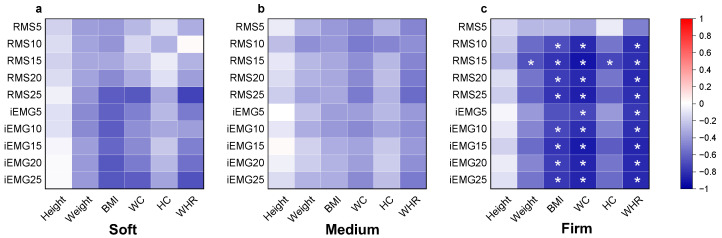
(**a**–**c**) Pearson correlation heatmap between participant anthropometric measurements and muscle activation across different mattresses on the lower back. Note: WC: waist circumference; HC: hip circumference; WHR: waist-to-hip ratio (WHR); *RMS*5, *RMS*10, *RMS*15, *RMS*20, and *RMS*25 represent the root mean square (*RMS*) values of muscle activation within successive 5 min intervals (0–5 min, 5–10 min, 10–15 min, 15–20 min, and 20–25 min, respectively). Similarly, *iEMG* values are calculated over the same intervals to assess muscle activation and frequency characteristics across time. Significant correlations after FDR correction are indicated by * for FDR-adjusted *p* < 0.05.

According to Figure 4, for the lower back, no statistically significant correlations were observed under soft and medium-firm mattress conditions. However, BMI, waist circumference (WC), and waist-to-hip ratio (WHR) were significantly negatively correlated with *RMS* and *iEMG* values across almost all time periods on the firm mattress. This suggests that individuals with higher BMI, larger waist circumferences (WC), and higher waist-to-hip ratios (WHR) experienced reduced muscle activity and greater relaxation. Body weight and hip circumference (HC) were negatively correlated with *RMS* values during the 10–15 min period, indicating that individuals with higher body weight and larger hip circumference may also experience greater relaxation on firm mattresses during specific time intervals.

### 3.4. Electromyographic Comparison Study on Mattresses with Three Different Firmness Levels

To investigate the effects of mattress firmness on muscle activation, we measured the activation levels of four muscles on soft and medium-firm mattresses.

**Figure 5:**
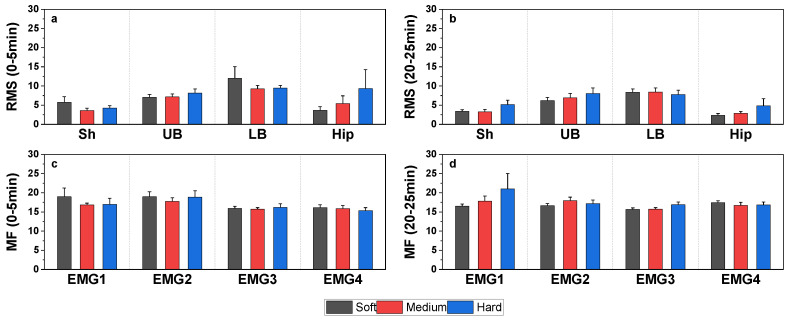
(**a**,**b**) Comparison of *RMS* values across different mattresses during the first and last five minutes. (**c**,**d**) Comparison of *MF* values across different mattresses during the first and last five minutes. Note: Sh: Shoulder (Upper Trapezius Descendens); UB: Upper Back (Lower Trapezius Descendens); LB: Lower Back (Erector Spinae, Iliocostalis); Hip: Hip (Gluteus Medius). All data are presented as mean ± SEM, *n* = 11.

Muscle activity during the initial 5 min and the final 5 min of the experiment was compared across all three mattresses (Figure 5). However, no significant differences in *RMS* or *MF* were found, suggesting that mattress firmness does not significantly influence muscle activation levels.

### 3.5. Discomfort Ratings

The repeated measures ANOVA results showed no significant differences in discomfort scores for the shoulder and upper back across the three mattress firmness levels. However, lower back discomfort scores differed significantly (F (2,20) = 5.844, *p* = 0.010, partial η^2^ = 0.369). Post hoc Newman–Keuls tests indicated that lower back discomfort on the firm mattress was significantly higher than on the medium-firm mattress (Table 3).

Although hip discomfort scores showed a significant difference (*p* = 0.049), post hoc tests did not identify any significant differences between the groups.

## 4. Discussion

This study aimed to investigate the effects of time, mattress firmness, and anthropometric parameters on sEMG activity during supine rest. The results showed no significant changes in sEMG activity across the time intervals (0–5, 5–10, 10–15, 15–20, and 20–25 min) on soft, medium, or firm mattresses. Neither the expected increase in lumbar fatigue over time nor significant differences in muscle activity between mattress types were observed. Further analysis revealed that sEMG activity during supine rest was primarily influenced by individual anthropometric parameters rather than time or mattress firmness.

When comparing the pressure at the mattress–human interface, the results showed that pressure increased with mattress firmness, as evidenced by more red pressure areas and a smaller contact area. The soft mattress had the largest contact area, while the firm mattress caused the lumbar region to suspend due to insufficient conformity. This suggests that mattress firmness affects both contact area and support characteristics [13,41,42].

Correlation analysis showed that muscle activation in the upper back has no correlation with anthropometric parameters, while lower back muscle activation had significant associations, especially on the firm mattress. The upper back muscles rely more on a natural relaxed state rather than external support [43], and the small fluctuations in sEMG signals at rest may explain the weaker correlation. In contrast, the lower back bears more body weight during sleep [40,44], making it more responsive to mattress support characteristics.

The medium-firm mattress demonstrated certain advantages. The sEMG activity on the medium-firm mattress remained more consistent across different time periods. In terms of subjective discomfort, the lumbar discomfort score on the firm mattress was significantly higher than on the medium-firm mattress, suggesting that the medium-firm mattress is more comfortable than the firm one. These findings highlight the advantages of the medium-firm mattress.

This result is consistent with previous studies, which have found the advantages of medium-firm mattresses in relieving lower back pain and improving sleep comfort, quality, and spinal alignment. This characteristic should be a key consideration in future mattress design [24,45,46].

Unlike traditional research, this study found that the relationship between mattress firmness and anthropometric parameters is not a simple linear one and should not rely solely on BMI to classify individuals. The use of BMI in previous mattress studies has been criticized for not considering body composition and distribution. As a result, recent studies have included waist-to-hip ratio (WHR) and waist circumference (WC) as additional indicators of body weight distribution [47,48]. In this study, BMI, WC, and WHR were negatively correlated with *RMS* and *iEMG* on firm mattresses; this is related to less muscle fatigue and a more relaxed muscle state [23,49]. This may be because, on firm mattresses, lumbar muscle fatigue is primarily caused by excessive support that leads to lumbar suspension. Individuals with larger waists, which are closer in size to their hips, may have a larger contact area at the waist, reducing lumbar spine curvature and minimizing muscle activation and fatigue. This suggests that individuals with higher BMI and WC tend to have more relaxed muscles, showing better adaptability to changes in mattress firmness.

Similarly, individuals with higher body weight also showed a negative correlation with *RMS* during certain time periods, suggesting that individuals with higher body weight may require firmer support to reduce muscle fatigue [50,51]. Similar conclusions have been drawn in studies on spinal alignment. Research by Shore, H. et al. showed that individuals with higher body weight have better spinal alignment when sleeping on firmer mattresses, while taller individuals find that a medium mattress helps keep the spine in a more neutral position [52].

This suggests that mattress design should consider individual differences, particularly matching support characteristics to anthropometric parameters, to better meet the needs of various body types. Waist circumference (WC) and waist-to-hip ratio (WHR) provide valuable data for predicting an individual’s adaptability to mattress firmness, with individuals of larger body sizes being better suited to firmer mattresses.

Although fluctuations in *RMS* and *MF* were observed, they did not reach statistical significance. These findings align with previous research, which found no significant differences in muscle activity across different mattress firmness levels [34]. This study focused on sEMG activity during rest, where small fluctuations in muscle activity may not reach statistical significance. Additionally, the small sample size of participants may have influenced the results. It is important to note that mattress interaction is a dynamic process involving posture changes, such as transitioning from supine to lateral positions, which can significantly affect overall sleep comfort [43].

## 5. Conclusions

This study systematically examined the effects of time, mattress firmness, and anthropometric factors on sEMG activity during supine rest. The results showed that, despite some fluctuations in sEMG indicators (such as *RMS* and *MF*) across different mattress types and time periods, these changes were not statistically significant, suggesting that muscle activity during rest remained relatively stable. Further analysis revealed significant correlations between sEMG activity and individual anthropometric parameters, with no noticeable direct impact from time or mattress firmness. This suggests that muscle activity in the supine position is influenced more by individual body characteristics than by mattress firmness alone.

The analysis revealed no significant correlation between upper back muscle activation and anthropometric factors, which may be attributed to the fact that upper back muscles rely more on a natural relaxed state rather than the support provided by the mattress. In contrast, for lower back muscles, on firm mattresses, BMI, WC, and WHR were negatively correlated with *RMS* and *iEMG*, suggesting that individuals with larger body sizes or wider waist circumferences are better able to adapt to changes in mattress firmness, exhibiting lower sEMG activity, possibly due to more evenly distributed support.

These findings underscore the importance of considering individual physiological characteristics in mattress design, particularly optimizing support based on BMI, WC, WHR, and body weight to better accommodate diverse body types. Additionally, the medium-firm mattress demonstrated the least fluctuation in sEMG activity and higher subjective comfort ratings, indicating a better balance between support and comfort. This study was limited to the analysis of sEMG activity during supine rest, with a small sample size consisting only of young, healthy individuals, which may limit the generalizability of the findings. Additionally, this study focused solely on the supine position. Different body postures during sleep may elicit distinct electromyographic responses. For instance, side-sleeping may engage muscles in the back, shoulders, and neck, while supine sleeping primarily involves the activation of lower back muscles. Therefore, investigating muscle activity across multiple sleep positions would provide a more comprehensive understanding of how mattress firmness affects muscle activity and sleep quality. Moreover, the recorded EMG signals in this study were relatively small, raising the possibility that they primarily reflect noise caused by pressure on the electrode–skin interface and electronic components rather than active muscle activity during rest.

Future research could benefit from including larger and more diverse samples to improve the generalizability of the results. Additionally, examining dynamic changes during sleep, such as side-sleeping and turning, would offer valuable insights. Expanding the analysis to include a wider range of variables and multidimensional parameters would further enhance our understanding of how mattress firmness impacts both sEMG activity and sleep quality.

## Figures and Tables

**Table 1:** Subjects Measures.

Title 1	Age (Years)	Height (m)	Mass (Kg)	BMI
Mean	23.09	1.72	63.64	21.41
Std. Deviation	1.86	0.81	7.15	1.42
Minimum	20.00	1.60	50.00	19.05
Maximum	26.00	1.86	75.00	24.22

**Table 2:** Comparison of pressure distribution for three mattresses.

	Soft	Medium	Firm	F	*p*	Partial η^2^
PP	59.36 ± 5.36 ^a^	47.60 ± 1.97 ^a^	134.48 ± 9.87 ^b^	186.18	<0.001	0.949
AP	11.56 ± 0.81 ^a^	15.08 ± 0.43 ^b^	33.03 ± 1.64 ^c^	67.42	<0.001	0.871
CA	2450.45 ± 75.69 ^a^	1958.34 ± 61.23 ^b^	1059.80 ± 44.40 ^c^	331.82	<0.001	0.971

Note: PP: Peak Pressure (mmHg); AP: Average Pressure (mmHg); CA: Contact Area (cm^2^). All parameters are presented as mean ± SEM, *n* = 11. Different superscript letters (e.g., a, b, c) indicate significant differences between groups (*p* < 0.05, Newman–Keuls post hoc test).

**Table 3:** Comparison of discomfort ratings for three mattresses.

	Soft	Medium	Firm	F	*p*	Partial η^2^
Shoulder	1.72 ± 0.60	0.91 ± 0.25	1.54 ± 0.43	1.09	0.356	0.098
UB	1.46 ± 0.67	1.27 ± 0.20	1.91 ± 0.71	0.50	0.616	0.096
LB	3.00 ± 0.81	0.91 ± 0.25 ^a^	3.36 ± 0.62 ^b^	5.84	0.010	0.369
Buttock	2.46 ± 0.81	0.91 ± 0.29	2.82 ± 0.67	3.53	0.049	0.261

UB: Upper back, LB: Lower back; All parameters are presented as Mean ± SEM, *n* = 11. Different superscript letters (e.g., a, b) indicate significant differences between groups.

## Data Availability

The datasets generated during the current study are available from the corresponding author on reasonable request at luobincl@bjfu.edu.cn.
